# Anterior Cruciate Ligament (ACL) Repair Using Dynamic Intraligamentary Stabilization Combined with Lateral Extra-articular Augmentation: A Case Report and Technical Note

**DOI:** 10.7759/cureus.68599

**Published:** 2024-09-04

**Authors:** Moritz Brunner, Jakob Hax, Louis Leuthard, Stefan Preiss, Michael Worlicek

**Affiliations:** 1 Department of Trauma Surgery, Regensburg University Medical Center, Regensburg, DEU; 2 Department of Knee and Hip Surgery, Schulthess Klinik, Zurich, CHE; 3 Department of Research and Development, Schulthess Klinik, Zurich, CHE

**Keywords:** isokinetic strength testing, acl repair, anterolateral ligament, patient-reported outcome, return to sport, dynamic intraligamentary stabilization, anterior cruciate ligament (acl)

## Abstract

The treatment options for an anterior cruciate ligament (ACL) rupture range from conservative therapy to ACL repair and reconstruction. ACL repair is particularly suitable for younger patients with an acute proximal tear, and moderate athletic demand. Preserving the ACL can restore its proprioceptive and stabilizing functions, avoid donor site morbidity, and shorten rehabilitation time. Repair techniques include the use of suture anchors, internal brace augmentation, and dynamic intraligamentary stabilization. Dynamic intraligamentary stabilization employs a coil spring mechanism for dynamic tibial fixation, allowing posterior translation during knee flexion, which stabilizes the ACL for optimal healing. However, patients with a positive preoperative pivot shift test have shown worse postoperative outcomes and higher failure rates after ACL repair. To address this, lateral extraarticular augmentation is recommended during ACL reconstruction to restore stability and prevent graft failure. We present the case of a 27-year-old female recreational handball player who sustained an acute proximal ACL rupture. Arthroscopic ACL repair was performed using the technique of dynamic intraligamentary stabilization combined with lateral extra-articular augmentation in the modified Lemaire technique. At six weeks postoperatively, the patient presented with a hard endpoint at Lachman's test and negative pivot shift. At five months, the isokinetic strength testing showed above-average strength values. At eight months, the patient underwent hardware removal and arthroscopically showed a fully healed ACL. For the final examination 12 months postoperatively, the patient presented with in-reference strength values in isokinetic strength testing and excellent scoring in patient-reported outcome measurements. The combination of dynamic intraligamentary stabilization and lateral extra-articular augmentation demonstrates an adequate treatment option for patients with proximal ACL ruptures and immediate functional demands in moderate activity level sports due to the rapid achievement of clinical and subjective stability as well as excellent results in isokinetic strength testing.

## Introduction

The spectrum of treatment for anterior cruciate ligament (ACL) rupture ranges from conservative therapy to ACL repair and ACL reconstruction [1]. Younger patients (25-45 years) with an acute ACL rupture (< 21 days), a proximal ACL tear configuration, and moderate athletic demand are especially suitable for ACL repair [2-4]. Since preservation of the ACL can restore both proprioceptive and stabilizing qualities of the ACL while preventing donor site morbidity and reducing the rehabilitation period, techniques range from ACL repair using suture anchors to internal brace augmentation to dynamic intraligamentary stabilization (DIS) [2,5,6].

Among the aforementioned techniques, DIS uses a coil spring mechanism for dynamic tibial fixation that allows posterior translation for any degree of flexion of the knee, resulting in stability for optimal healing of the ACL [2]. Sherman et al. found a worse postoperative outcome and higher failure rates in patients who underwent ACL repair and concomitantly had a positive preoperative pivot shift test [3]. To restore the pivot shift and to prevent graft failure, lateral extra-articular augmentation is an excellent choice in ACL reconstruction [7]. Thus, the hypothesis was that the combination of both techniques, DIS and lateral extra-articular augmentation, is a practical option for patients who are suitable for ACL repair but have a positive pivot shift test and risk of failure due to high levels of athletic demand. We present the case of a patient who underwent ACL repair using DIS combined with lateral extra-articular augmentation to restore knee stability and enable a fast return to pivoting sports.

## Case presentation

**Figure 1:**
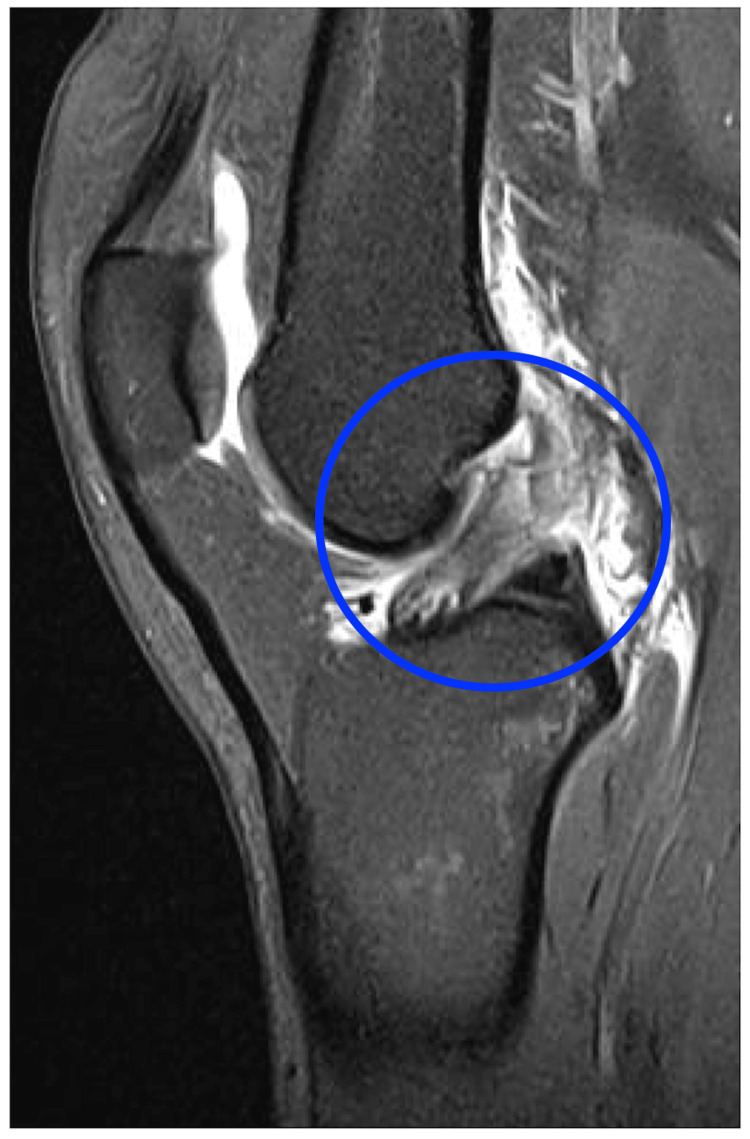
Preoperative MRI (sagittal view) of the right knee demonstrating a proximal anterior cruciate ligament rupture (blue circle).

A 27-year-old female recreational handball player presented to our clinic after suffering a knee distortion on the right side while competing in a handball game. Clinical examination revealed knee pain, moderate swelling, and limited range of motion (ROM) of 90-15-0°. The subjective feeling of instability was confirmed by a positive grade 2 Lachman test without a hard endpoint and a positive grade 2 pivot shift test. MRI confirmed a proximal ACL tear (Figure 1).

Owing to the young age, the recreational athletic demand as well as the instability, surgical treatment was recommended. Based on the proximal rupture morphology (Sherman type 1 tear, van der List type 2) and early timing, ACL repair using DIS was considered [3,8]. Additional extra-articular lateral augmentation using a modified LeMaire technique was recommended because of rotational instability and moderate to high athletic demand in pivoting sports. The patient was informed both verbally and in written form about the planned procedure and they consented to undergo surgery.

Surgical treatment 

ACL repair using DIS and lateral extra-articular augmentation was performed as previously described but with a combination of both techniques [2,9]. 

**Figure 2:**
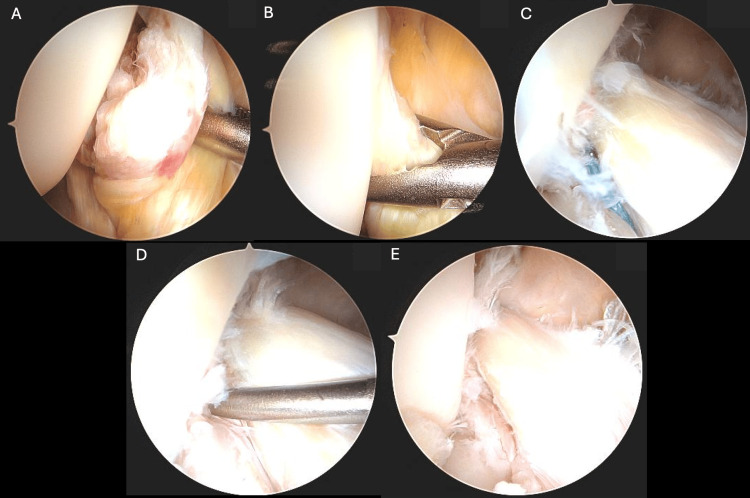
Arthroscopic views showing a femoral ACL tear with good mobilization of the ACL stump (A-B), sutures passed through the ACL (C), and continuous proximity of the two ligamentous stumps (D-E). ACL: anterior cruciate ligament

During spinal anesthesia, the patient was positioned in the standard arthroscopic setting and the right knee was sterilely prepared. A standard diagnostic arthroscopy was then conducted through an anterolateral portal wich revealed a directly femoral proximal ACL tear. The ACL stump could be mobilized easily and showed good tissue quality. In the figure of four position, the ACL could be well reduced with the grasping forceps, so that the indication for an ACL repair can be made (Figure 2A, 2B). 

Using an intra-articular guide, the tibial attachment point of the ACL was determined and a wire was passed through the tibia to reach this marked location. A 10.5-mm threaded sleeve (Mathys Ltd Bettlach, Bettlach, Switzerland) was then surgically placed in the tibia. A suture guide was then inserted through the threaded sleeve to access the distal ACL stump, where an initial suture was passed through the ligament (Figure 2C).

The femoral attachment site was located with a guide accessible through the anteromedial portal. A wire was passed at a 120-degree flexion angle to the lateral aspect of the femur. This wire was then threaded through the skin, and the final polyethylene wire was inserted from the anterolateral femoral position into the anteromedial tibial region. The stability of the wire was secured at the femoral attachment site with a hinged anchor.

**Figure 3:**
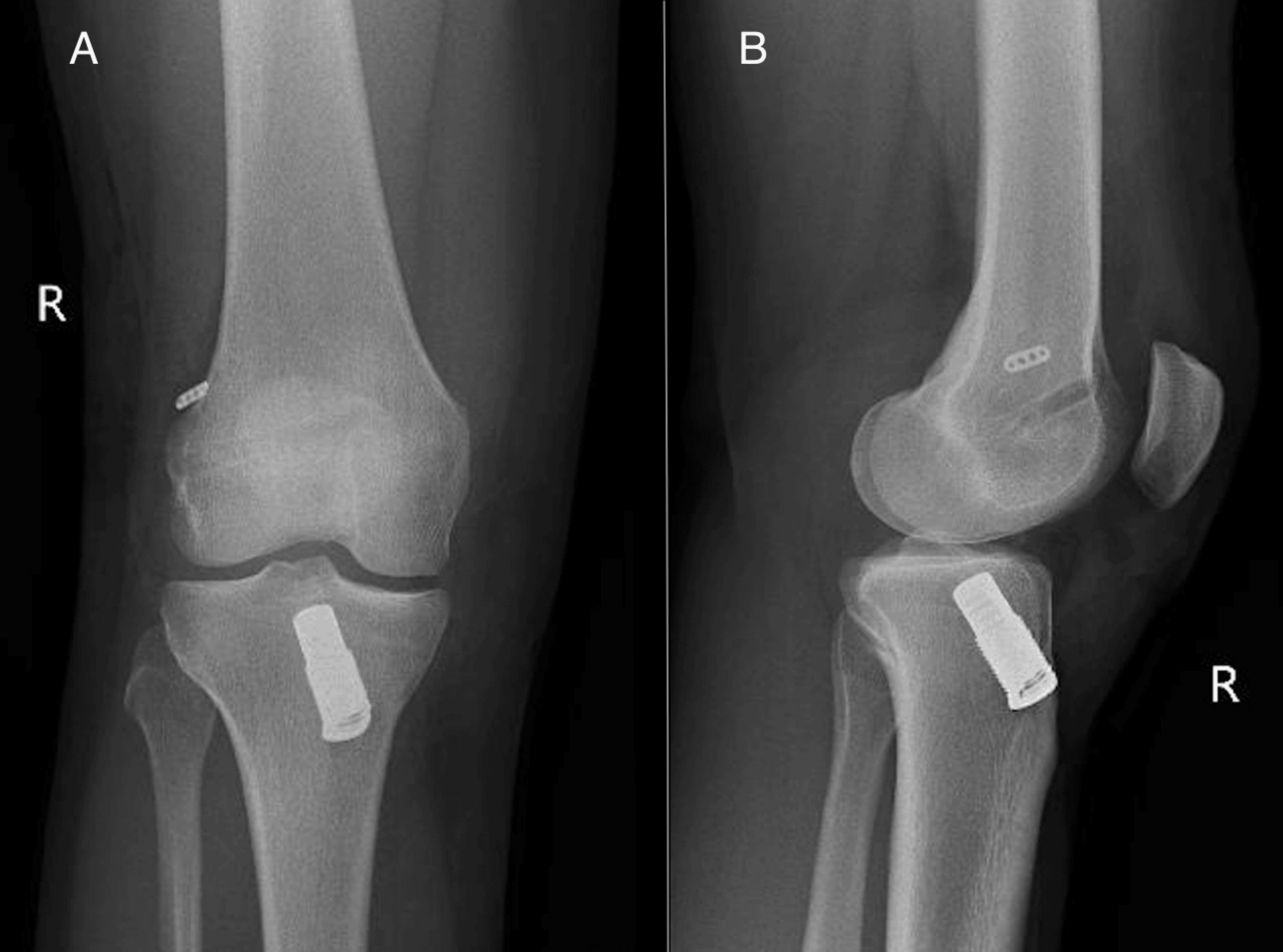
Postoperative anteroposterior (A) and lateral (B) radiographs of the right knee.

A metal spring, which can be seen in the postoperative radiographs (Figure 3A, 3B), was placed in the threaded sleeve, and the polyethylene wire was firmly secured with a cap at the end of the screw while maintaining a tension of 80 N. The DIS technique ensures consistent posterior translation of the knee in all degrees of flexion, guaranteeing the continuous proximity of the two ligamentous stumps throughout the procedure (Figure 2D, 2E). 

Now, we moved on to the anterolateral stabilization using the modified LeMaire technique. To begin, a skin incision was made starting from the lateral epicondyle and extending towards Gerdy's tubercle. Hemostasis was ensured, and the area involving the iliotibial tract was prepared. A strip measuring 1 cm in width and 8 cm in length was dissected from the transition between the dorsal and middle thirds, starting proximally and proceeding towards Gerdy's tubercle. The proximal end was reinforced with Vicryl sutures using a baseball suture technique.

Next, the lateral collateral ligament was identified, and an incision was made ventral to it. Using an overholt, the tractus strip was cut in the direction of the lateral epicondyle. The strip was located, and a wire was placed slightly proximal and dorsal to the lateral epicondyle in the ventral direction. A 5 mm drill bit was used to drill until just before reaching the opposite cortical bone. A shuttle thread was inserted at this point. A potential tunnel conflict can be avoided by advancing the scope into the femoral tunnel and visualizing the femoral tunnel.

With the help of the shuttle thread, the tractus strip was pulled into the femoral tunnel, ensuring it was held firmly under tension. In extension and a neutral position, a 5 x 23 mm interference screw was inserted. 

Postoperative rehabilitation included a knee brace for five days, partial weight bearing for three to four weeks, and functional physical therapy.

Outcome and follow-up 

**Figure 4:**
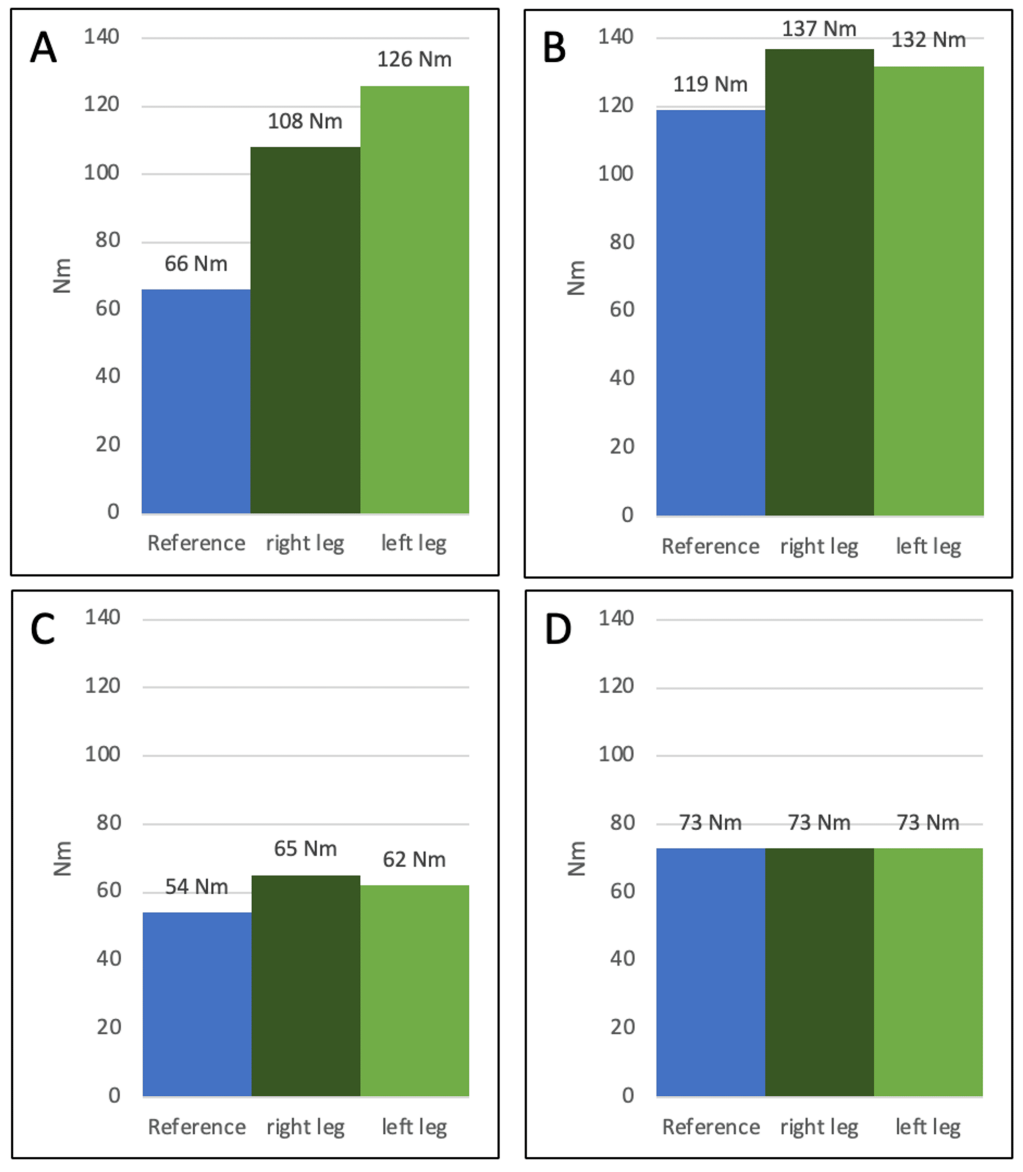
Isokinetic strength measurement of extensor and flexor muscles at five and 12 months postoperatively in comparison to reference strength values for extensor and flexor muscles. (A) Knee extension strength (Nm) five months postoperatively in relation to body weight (60 kg); (B) Knee extension strength (Nm) 12 months postoperatively in relation to body weight (60 kg); (C) Knee flexion strength (Nm) five months postoperatively in relation to body weight (60kg); (D) Knee flexion strength (Nm) 12 months postoperatively in relation to body weight (60 kg)

At six weeks postoperatively, the patient presented with a ROM of 0-3-115°, a stable anterior drawer, a Lachman test with a hard endpoint, and a negative pivot shift test. At five months postoperatively, the patient underwent isokinetic strength testing and showed above-average strength values for flexor and especially extensor muscles (Figure 4).

**Figure 5:**
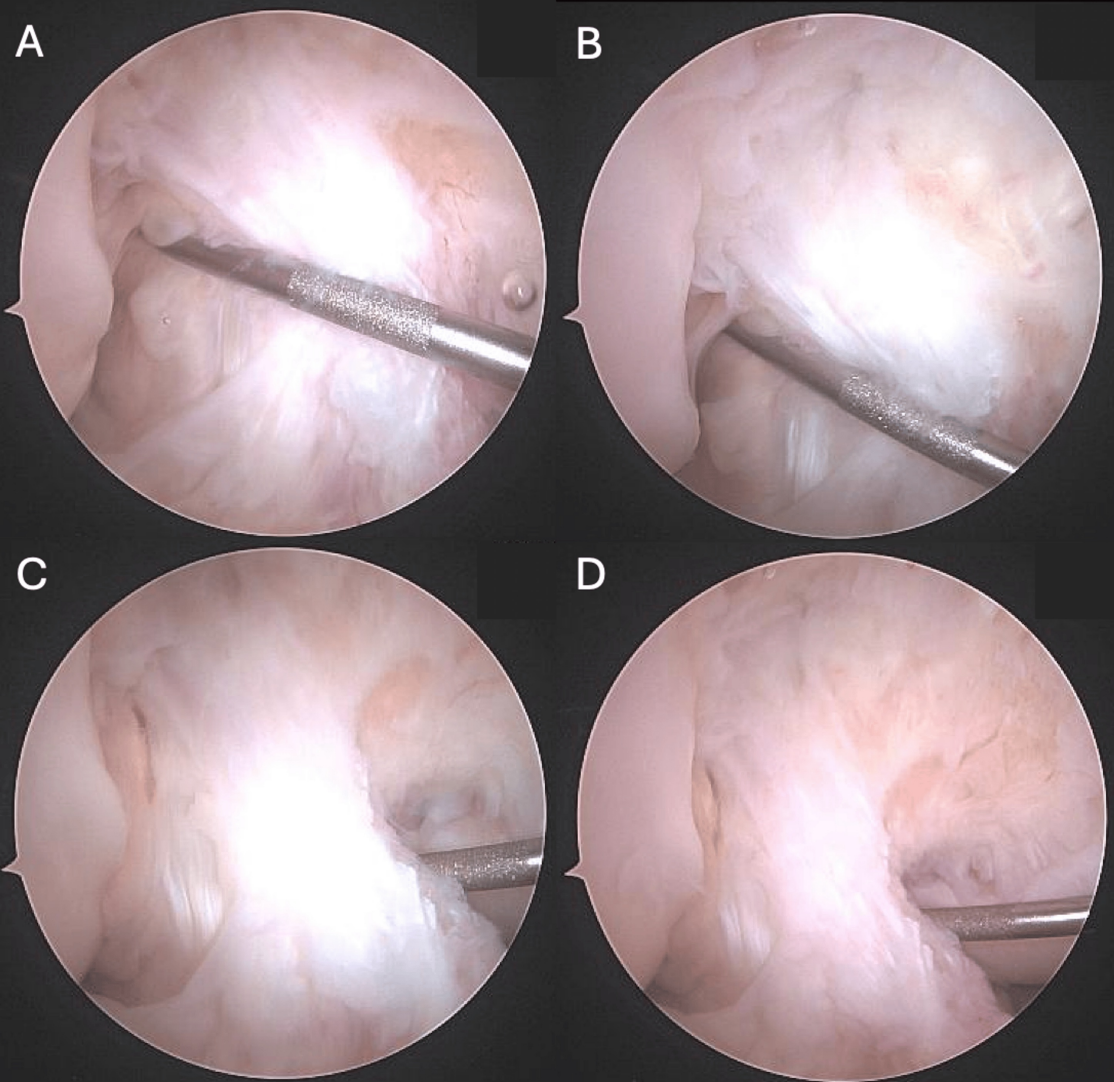
Arthroscopic views showing the fully healed ACL eight months postoperatively. ACL: anterior cruciate ligament

At eight months postoperatively, the patient underwent hardware removal and arthroscopically showed a fully healed ACL (Figure 5). For the final examination at 12 months postoperatively, the patient presented with a full ROM in reference strength values for extensor and flexor muscles (Figure 4) and excellent scoring in patient-reported outcome measurements (Table 1).

**Table 1:** Patient-reported outcome measurements. IKDC: International Knee Documentation Committee Subjective Knee Evaluation Form; COMI: Core Outcome Measures Index

Patient-reported outcome score	Preoperative	6 months	12 months
IKDC	32.18	83.91	98.85
COMI	5.95	1.75	0

## Discussion

In the current case, the combination of DIS and lateral extra-articular augmentation led to a fully healed ACL, both subjective and objective knee stability, excellent strength values, and a rapid return to sports. 

The first step, essential for the success of ACL preservation, is patient selection. Most important is the proximal tear configuration, which shows the best outcome after ACL repair. Sherman et al. classified ACL tears into four types: Type 1 shows a proximal avulsion with minimal tissue left on the femur, type 2 shows up to 25% and type 3 up to 33% tissue left on the femur, whereas type 4 shows a mid-substance rupture [3]. According to a study by van der List et al., approximately 40% of the ACL tears examined showed type 1 and type 2 tears, which are most suitable for ACL repair [3,5,8]. 

Furthermore, patient age is an important factor in selecting patients for ACL repair, specifically DIS. In the original publication of the DIS technique by Eggli et al., the appropriate patient age was defined as <45 years [2]. Glasbrenner et al. found younger patient age to be a risk factor for recurrent instability in both DIS and ACL reconstruction and recommended DIS for patients > 25 years old; therefore, our patient who was 27 years old, falls within the scientifically defined age range for DIS [4]. The indications and contraindications for DIS are given in Table 2.

**Table 2:** Indications and contraindications for DIS ACL: anterior cruciate ligament; DIS: dynamic intraligamentary stabilization

Indications	Contraindications
Proximal tear configuration	Mid substance tear
Good tissue quality	Bad tissue quality
Acute ACL ruptures (<21 days)	Chronic ACL ruptures (> 21 days)
Young patient age	High athletic demand
Moderate athletic demand	

Several studies identified physical activity and, especially, participation in competitive sports (Tegner activity scale > 7) as risk factors for DIS failure [4,10]. Although the Tegner activity scale was not assessed for our patient, we can assume a Tegner activity scale of 6 based on the sport she plays, handball [11]. 

Compared to the current gold standard, ACL reconstruction, successful ACL preservation offers several advantages. ACL repair prevents donor site morbidity, such as anterior knee pain, which is correlated with loss of ROM and reduced strength [12]. At the same time, by preserving the native ACL, the knee is given the opportunity to maintain its proprioception, which is mediated by the ACL and would be lost by performing an ACL reconstruction using auto- or allograft [13,14]. 

The technique that was chosen for ACL preservation in the current case was DIS. The main challenge was to recreate an optimal environment for the healing process of the ACL. DIS combines ACL repair with intraligamentary stabilization, which enhances the healing process by a constant posterior translation in any degree of flexion through the dynamic screw spring mechanism unlike a simple augmentation, e.g. with suture augmentation [2,5,15]. Several clinical studies reported good clinical short to midterm results regarding failure rate, patient satisfaction, premature rupture of membranes (PROM), knee laxity, and return to sports [2,4,16-18]. 

However, the indication for DIS should be very specific and fulfill all the requirements of the patient selection prior discussed in order to keep failure and recurrent instability to a minimum [2,4,10]. Failure rates between 15-60% have been reported in various studies [4,10,18]. Another disadvantage is the need for a second operation to remove the coil spring system. 

In the study by Sherman et al., an increased preoperative pivot shift resulted in poor postoperative outcomes [3]. This and the findings from studies investigating ACL reconstruction and other forms of ACL repair in combination with lateral extraarticular augmentation led to the rationale of combining the technique of ACL repair using DIS with lateral extraarticular augmentation [7,19]. These studies showed not only significantly lower failure rates for patients undergoing additional lateral extra-articular augmentation, but Ferretti et al. also found non-inferior knee laxity, graft maturity, PROMs, and a significantly shorter duration to return to the preinjury level of sport while comparing ACL repair and anterolateral structure repair to ACL reconstruction and lateral extra-articular tenodesis [7,19]. 

## Conclusions

In this case, combining DIS with lateral extra-articular augmentation led to a fully healed ACL, a stable knee, excellent strength, and a quick return to sports. Successful ACL preservation depends critically on selecting patients with a proximal tear configuration. Patient age is also crucial, with the 27-year-old patient of the current case fitting the ideal age range. DIS merges ACL repair with intraligamentary stabilization, offering constant posterior translation during knee flexion through a dynamic screw spring mechanism, unlike simple suture augmentation. Specific indications for DIS are necessary to reduce failure and recurrent instability, which justified our choice to combine DIS with lateral extra-articular augmentation.
